# Wideband and Channel Switchable Mode Division Multiplexing (MDM) Optical Power Divider Supporting 7.682 Tbit/s for On-Chip Optical Interconnects

**DOI:** 10.3390/s23020711

**Published:** 2023-01-08

**Authors:** Tun-Yao Hung, Guan-Hong Chen, Yuan-Zeng Lin, Chi-Wai Chow, Yin-He Jian, Pin-Cheng Kuo, Ching-Wei Peng, Jui-Feng Tsai, Yang Liu, Chien-Hung Yeh

**Affiliations:** 1 Department of Photonics & Graduate Institute of Electro-Optical Engineering, College of Electrical and Computer Engineering, National Yang Ming Chiao Tung University, Hsinchu 30010, Taiwan; 2 Department of Photonics & Graduate Institute of Electro-Optical Engineering, College of Electrical and Computer Engineering, National Chiao Tung University, Hsinchu 30010, Taiwan; 3 Philips Electronics Ltd., N.T., Hong Kong; 4 Department of Photonics, Feng Chia University, Taichung 40724, Taiwan

**Keywords:** silicon photonics (SiPh), mode division multiplexing (MDM), orthogonal frequency-division multiplexing (OFDM), optical interconnect

## Abstract

Silicon photonics (SiPh) are considered a promising technology for increasing interconnect speed and capacity while decreasing power consumption. Mode division multiplexing (MDM) enables signals to be transmitted in different orthogonal modes in a single waveguide core. Wideband MDM components simultaneously supporting wavelength division multiplexing (WDM) and orthogonal frequency-division multiplexing (OFDM) can significantly increase the transmission capacity for optical interconnects. In this work, we propose, fabricate and demonstrate a wideband and channel switchable MDM optical power divider on an SOI platform, supporting single, dual and triple modes. The switchable MDM power divider consists of two parts. The first part is a cascaded Mach–Zehnder interferometer (MZI) for switching the data from their original TE_0_, TE_1_ and TE_2_ modes to different modes among themselves. After the target modes are identified, mode up-conversion and Y-branch are utilized in the second part for the MDM power division. Here, 48 WDM wavelength channels carrying OFDM data are successfully switched and power divided. An aggregated capacity of 7.682 Tbit/s is achieved, satisfying the pre-forward error correction (pre-FEC) threshold (bit-error-rate, BER = 3.8 × 10^−3^). Although up to three MDM modes are presented in the proof-of-concept demonstration here, the proposed scheme can be scaled to higher order modes operation.

## 1. Introduction

In recent years, high bandwidth demands have been due to the worldwide deployment of the Internet-of-Things (IOT), big data analysis, on-line gaming and shopping, video streaming, and many different internet-based services [1,2,3,4,5,6,7]. For the data center networks, the electronic interconnect data rates are limited by high power consumption. Hence, silicon photonics (SiPh) are considered a promising technology for increasing interconnect speed and capacity while decreasing the power consumption [8,9,10]. SiPh devices are fabricated using mature complementary metal oxide semiconductor (CMOS) fabrication technologies; hence, high yield and efficient SiPh devices can be implemented [11,12,13,14]. To enhance the SiPh interconnect transmission capacity, different technologies have already been proposed, such as wavelength division multiplexing (WDM) [15,16], polarization division multiplexing (PolDM) [17,18] and spatial division multiplexing (SDM) [19,20], as well as advanced digital multiplexing schemes, e.g., orthogonal frequency-division multiplexing (OFDM) [21], non-orthogonal multiple access (NOMA) [22], etc. Mode division multiplexing (MDM) [19] is a popular way to achieve SDM, which allows signals to be propagated in different orthogonal modes in a single waveguide core. MDM systems using few-mode optical fiber (FMF) were also reported [23,24]. Different fiber-based or free-space optics-based MDM multiplexer/demultiplexers (Mux/Demux) were demonstrated, including those utilizing directional mode selective couplers [25], long period fiber grating (LPFG) [26], free-space optics [27], and photonic lanterns (PLs) [28].

Alongside the fiber-based and free-space optics-based MDM Mux/Demux, SiPh based MDM Mux/Demux is easier to realize as different modes transmitting in a waveguide core can be easily preserved and converted in the planar waveguide structures. Using an asymmetrical directional coupler (ADC) is a sample way to achieve wideband, higher conversion efficiency and low crosstalk MDM Mux/Demux on a silicon-on-insulator (SOI) platform [19,20]. Wideband MDM components, simultaneously supporting WDM and OFDM, can significantly increase the transmission capacity for optical interconnects. A power divider is one of the basic building blocks for SiPh on-chip networks; and dual-mode and triple-mode on-chip optical power dividers were successfully demonstrated recently [29,30,31]. These MDM building blocks can allow the realization of future multi-mode photonic integrated circuits (PICs) [32]. However, traditional on-chip optical components usually support single-mode operation.

In this work, we propose, fabricate and demonstrate a wideband and channel switchable MDM optical power divider on an SOI platform, supporting single, dual and triple modes. The switchable MDM power divider consists of two parts. The first part is a cascaded Mach–Zehnder interferometer (MZI) for switching the data from their original transverse-electric (TE) modes, TE_0_, TE_1_ and TE_2_, to different modes among themselves. After the target modes are identified, mode up-conversion and Y-branch are utilized in the second part for the MDM power division. Here, 48 WDM wavelength channels carrying OFDM data are successfully switched and power divided. An aggregated capacity of 7.682 Tbit/s is achieved, satisfying the pre-hard-decision forward-error-correction (pre-HD-FEC) threshold (bit-error-rate, BER = 3.8 × 10^−3^). Although up to 3 MDM modes are presented in this proof-of-concept demonstration here, the proposed scheme can be scaled to higher order modes operation.

## 2. Design and Simulation

**Figure 1:**
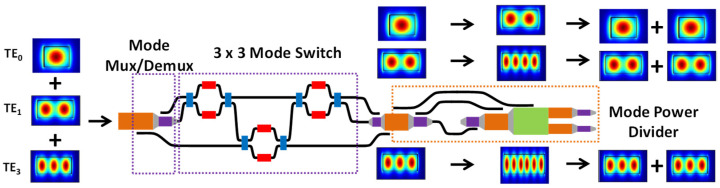
Design architecture of our proposed wideband and channel switchable MDM optical power divider. Insets: cross sections of different orders of MDM modes.

Figure 1 shows the design architecture of our proposed wideband and channel switchable MDM optical power divider. It consists of two parts. The first part is a cascaded MZI for switching the data from their original TE_0_, TE_1_ and TE_2_ modes to different modes among themselves. After the target modes are identified, mode up-conversion and Y-branch are utilized in the second part for the MDM power division. The insets in Figure 1 illustrate the cross sections of different orders of MDM modes.

**Figure 2:**
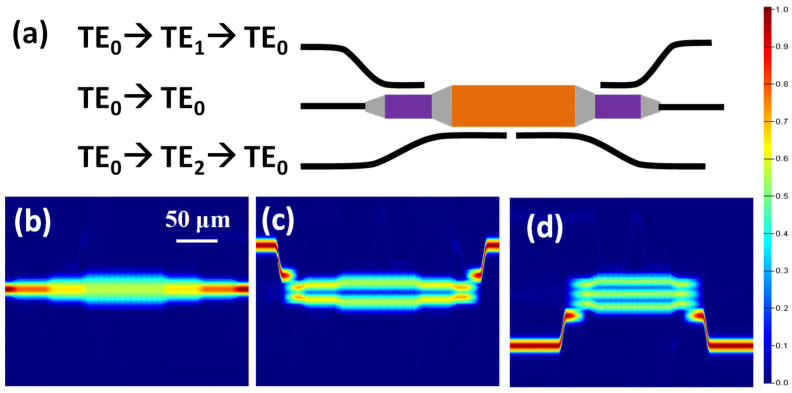
(**a**) Schematic of the SOI based ADC MDM Mux/Demux. FDTD simulation result of (**b**) TE_0_ to TE_0_, (**c**) TE_0_ to TE_1_ and then back to TE_0_, (**d**) TE_0_ to TE_2_ and then back to TE_0_.

Before discussing the operation mechanism of the MDM optical power divider, we first discuss how to use the ADC for combining the TE_0_, TE_1_ and TE_2_ modes in a single bus waveguide. Figure 2a shows the structure schematic of the ADC-based MDM Mux/Demux. As discussed in [19], an ADC structure consists of a narrower access waveguide (for the TE_0_ mode transmission) and a wider bus waveguide (for the high order modes transmissions). When the access waveguide and bus waveguide are close to each other, the fundamental TE_0_ mode can be converted to higher order modes, or vice versa when the phase-matching condition is satisfied, as discussed in the couple mode theory. As the whole proposed MDM power divider is on an SOI platform, the ADC structure is no exception. The dimensions of the silicon access waveguide are 0.45 μm × 0.22 μm. The buried oxide layer (BOX) is 2 μm, and the ADC gap in the coupling region is 0.15 μm. Here, we used an Ansys Lumerical^®^ finite-difference time-domain (FDTD) technique to simulate the ADC-based MDM Mux/Demux. As discussed before, the access waveguide has a width of 0.45 μm supporting the TE_0_ mode. In order to meet the phase matching condition, the bus waveguide widths are 0.932 μm and 1.416 μm, supporting the TE_1_ and TE_2_ modes, respectively. Furthermore, the coupling lengths for the TE_0_-TE_1_ and TE_0_-TE_2_ modes ADC are 17 μm and 22.5 μm, respectively. Figure 2b–d show the FDTD simulation results when light at the fundamental TE_0_ mode is inputted from the left-hand side and outputted at the right-hand side. As shown in Figure 2b, the MDM Mux/Demux can maintain the TE_0_ mode at the device output. Figure 2c,d illustrate that the fundamental TE_0_ mode can be up-converted to TE_1_ and TE_2_, and then back to the TE_0_ mode again at the output. The simulation results also reveal that the coupling efficiencies of ADC in all the channels are more than 95%.

In the 3 × 3 mode switch, the data carried in the TE_0_, TE_1_, and TE_2_ modes can be converted among one other. It has a similar structure to that reported in [33]. As shown in Figure 1, it consists of three cascaded 2 × 2 MZIs. Each MZI consists of two symmetrical arms with thermal-optic phase shifters and two multimode interferometers (MMI). In order to provide wideband operation, the path difference between the two arms of the MZI is zero. The thermo-optic phase shifter is based on a p-doped waveguide, which is used as a resistive heater to provide the phase shifting. Its characteristics will be discussed in the next section. In order to balance the doping-induced optical loss, the thermo-optic phase shifters are utilized in both arms of the MZI, but only one arm has the electrical contact for applying the external electrical bias.

**Figure 3:**
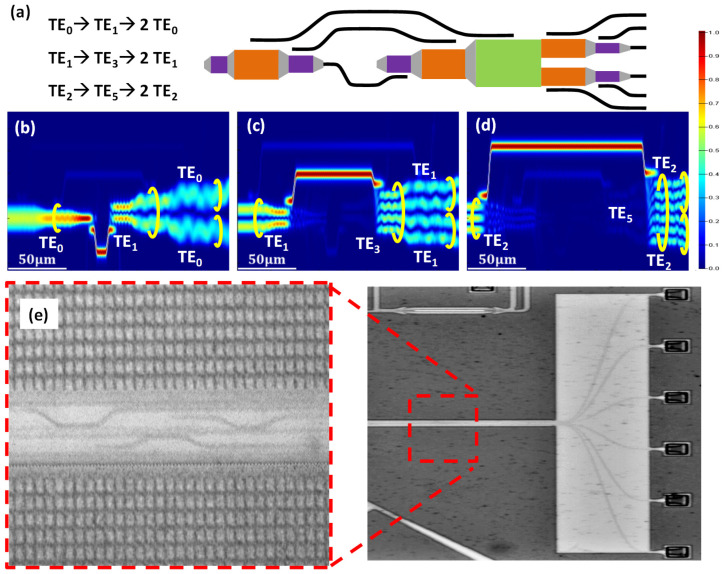
(**a**) Schematic of the SOI-based MDM optical power divider. FDTD simulation result of the MDM optical power divisions at (**b**) TE_0_, (**c**) TE_1_, (**d**) TE_2_. (**e**) Photograph of the MDM optical power divider, showing the magnified section of the ADCs for mode conversions.

Finally, we discuss the operation mechanism of the MDM optical power divider. Figure 3a shows the schematic of the SOI-based MDM 3-dB power splitter structure. The operation principle is based on the mode up-conversions using ADCs, and then the higher order modes are split by the Y-bench into two TM_2n−1_ modes, where n is an integer. Figure 3b–d show the FDTD simulation results of the MDM optical power divisions at TE_0_, TE_1_, and TE_2_ modes, respectively, launched from the left-hand side and outputted at the right-hand side. Without loss of generality, we use the TE_0_ mode as an example. If the TE_0_ mode needs to be power divided, it will be mode up-converted to the TE_1_ mode before power division, as shown in Figure 3b. Then, two equal power TE_0_ modes can be produced at the output of the Y-bench. Similarly, the TE_1_ and TE_2_ are mode up-converted to the TE_3_ and the TE_5_ mode through the ADCs. Then, the Y-bench would split TE_3_ and TE_5_ modes to two 50% power TE_1_ and TE_2_ modes, as shown in Figure 3c,d. In the experiment, since we cannot measure the output TE_1_ and TE_2_ modes of the MDM power divider directly, they will be further converted to TE_0_ modes via ADCs, as illustrated in Figure 3a. As a result, there are six outputs from the MDM optical power divider. Figure 3e shows the photograph of the MDM optical power divider, showing the magnified section of the ADCs for mode conversions, and the six TE_0_ outputs at the right part of the device.

## 3. Experiment, Results and Discussion

**Figure 4:**
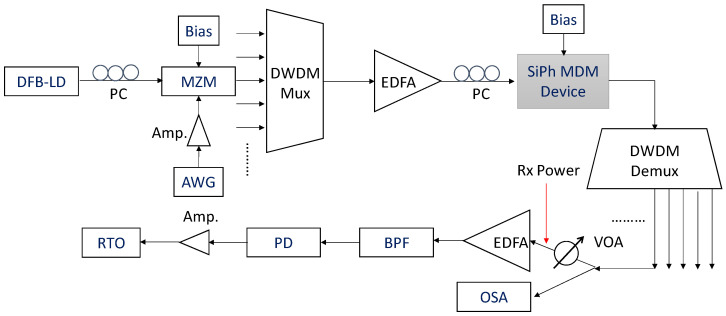
Experimental setup to evaluate the proposed switchable MDM optical power divider. AWG: arbitrary waveform generator; MZM: Mach–Zehnder modulator; DFB-LD: distributed feedback laser diode; EDFA: erbium-doped optical fiber amplifier; Amp.: electrical amplifier; OSA: optical spectrum analyzer; VOA: variable optical attenuator; BPF: optical band-pass filter; PD: photodiode; RTO: real-time oscilloscope.

The proposed switchable MDM optical power divider was fabricated by IMEC^®^. The experimental setup to evaluate the proposed switchable MDM optical power divider is shown in Figure 4. We use the OFDM signal with 500 data lengths, 512 fast-Fourier transform (FFT) size, 170 subcarriers and 32 cyclic prefix (CP) length to evaluate the system. The OFDM signal is generated from an arbitrary waveform generator (AWG, Tektronix^®^ AWG 70001). The OFDM modulation includes the serial-to-parallel (S/P) binary data conversion; symbol mapping, inverse fast-Fourier transform (IFFT), parallel-to-serial (P/S), CP insertion. The electrical OFDM signal is inputted to a 40-GHz bandwidth Mach–Zehnder modulator (MZM) via an electrical amplifier (Amp.) to modulate the optical signal, which is generated from different wavelength distributed feedback laser diodes (DFB-LDs). Different dense wavelength division multiplexed (DWDM) OFDM optical signals are combined via a DWDM Mux, and they are amplified by an erbium-doped optical fiber amplifier (EDFA) to compensate the transmission losses. The optical signals are coupled into the proposed SiPh-based MDM optical power divider via an on-chip grating coupler (GC). Electrical signals are applied to the 3 × 3 mode switch via a radio frequency (RF) probe for the different mode switching. Then, the optical power divided signals are coupled out of the chip, also via on-chip GC. DWDM Demux is used to separate different DWDM channels. Optical spectrum analyzer (OSA) is used to measure the operation wavelength window of the device. A variable optical attenuator (VOA) is used to control the received power, before launching into the optical pre-amplified receiver (Rx). An optical band-pass filter (BPF) is used to remove the out-of-band amplified spontaneous emission (ASE) noise from the pre-amplified EDFA. Finally, a 40-GHz bandwidth photodiode (PD) is used to receive the optical signal, and an 80 GS/s real-time oscilloscope (RTO, LeCroy^®^ 816ZI-B) is used to capture the waveform for the OFDM demodulation. The demodulation includes re-sampling, data synchronization, removal of CP, S/P, FFT, scaling of power, de-mapping of symbol, and BER calculation.

**Figure 5:**
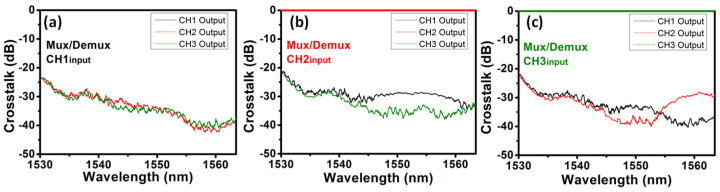
Measured normalized mode crosstalk of (**a**) MDM CH1, (**b**) MDM CH2 and (**c**) MDM CH3.

We first experimentally evaluate the ADC for mode Mux, and Demux the TE_0_, TE_1_ and TE_2_ modes. Figure 5a–c show the experimentally measured normalized mode crosstalk of the MDM Mux and Demux when the broadband light source is launched at Channel 1 (CH1) to Channel 3 (CH3) of MDM, respectively. We normalized the optical spectra measured at the three output ports with the corresponding output port spectrum. Taking CH1 for an example, the optical spectra at the CH2 and CH3 output ports are normalized with the optical spectrum at the CH1 output port when the light is launched into the CH1 input port. As a result, the optical spectrum at the corresponding output port becomes united (i.e., 0 dB), and the optical spectra at other output ports reveal the mode crosstalk. It can be observed that the typical mode crosstalk is low, which is <−30 dB in most of the wavelength windows within the C-band.

**Figure 6:**
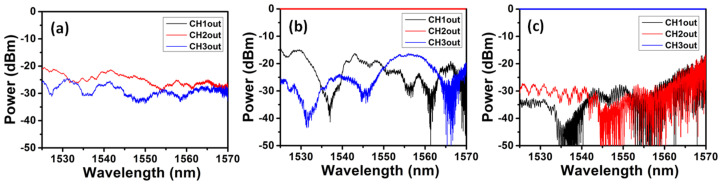
Measured normalized crosstalk observed at different output ports of the 3 × 3 switch when inputted at (**a**) CH1, (**b**) CH2 and (**c**) CH3.

We first experimentally characterize a single 2 × 2 switch. Table 1 shows the applied bias voltage, measured current drawn, and calculated resistance, as well as power consumption by the switch. The calculated resistances of the thermo-optic phase shifter are similar when the applied bias voltage increases from 5 V to 20 V, and the calculated resistance is about 1.3 kΩ. We also measure that a bias voltage of 12 V is needed to change the state of the switch from bar to cross, which requires 0.1 W electrical power. Figure 6a,b show the experimentally measured normalized crosstalk observed at different output ports of the 3 × 3 switch when inputted at CH1 to CH3, respectively. Here, CH1 is defined as the input port being the TE_0_ mode port for MDM and output from the top port of the 3 × 3 switch, as shown in Figure 1, CH2 is defined as the input port being the TE_1_ mode port for MDM and output from the middle port of the 3 × 3 switch, and CH3 is defined as the input port being the TE_2_ mode port for MDM and output from the bottom port of the 3 × 3 switch. Similar to the measured optical spectra in Figure 5a–c, the optical spectra at the three output ports are normalized with respect to the corresponding input channel, respectively. We can observe that the typical switch crosstalk is low enough, which is <−20 dB in most of the 45 nm wavelength windows.

**Figure 7:**
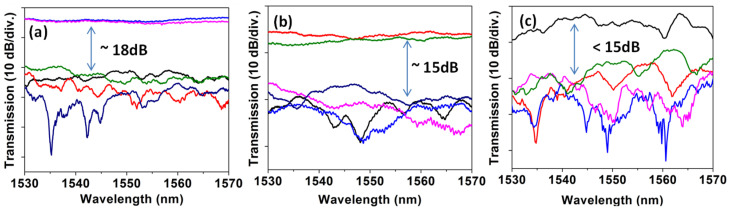
Measured relative crosstalk of the MDM power divider for power divisions of (**a**) TE_0_, (**b**) TE_1_, (**c**) TE_2_ modes.

Then, we experimentally evaluate the MDM optical power divider. As illustrated in Figure 3a, the MDM power divider can support three operations: “TE_0_ → TE_1_ → 2 TE_0_”, “TE_1_ → TE_3_ → 2 TE_1_”, and “TE_2_ → TE_5_ → 2 TE_2_”. In the experiment, since we cannot measure the output TE_1_ and TE_2_ modes of the MDM power divider directly, they will be further converted to TE_0_ modes via ADCs as also illustrated in Figure 3a. As a result, there are six outputs from the MDM optical power divider. Figure 7a–c show the experimentally measured relative crosstalk of the MDM power divider for power divisions of TE_0_, TE_1,_ TE_2_ modes, respectively. We can observe ~−18 dB crosstalk for the TE_0_ power division, and ~−15 dB crosstalk for the TE_1_ and TE_2_ power divisions, respectively. The higher crosstalk in the TE_1_ and TE_2_ power divisions could be due to more instances of mode conversions as well as fabrication error. The overall loss of the MDM power divider is ~5 dB for the TE_0_ mode and ~10 dB for higher order modes.

**Figure 8:**
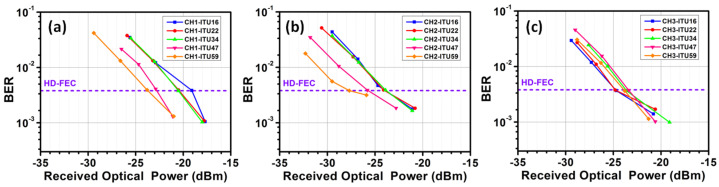
Measured BER curves of some selected wavelength channels of the 3 × 3 modes switch (**a**) CH1, (**b**) CH2 and (**c**) CH3.

Then, we evaluate the BER performance of the 48 DWDM wavelength channels in C-band. The BER performances of the outputs of the 3 × 3 switch are shown in Figure 8a–c, respectively. Here, we evenly chose five wavelength channels for display: 1530.33 nm (ITU-16), 1539.77 nm (ITU-22), 1550.12 nm (ITU-34), 1559.79 nm (ITU-47) and 1564.68 nm (ITU-59). Measurement results show that every wavelength channel can satisfy the pre-HD-FEC threshold. The target of our BER measurement is to achieve the highest possible OFDM transmission data rate while satisfying the HD-FEC threshold. Therefore, wavelength channels with higher Rx sensitivities do not necessarily mean poorer performance, as these channels may carry higher data rates.

**Figure 9:**
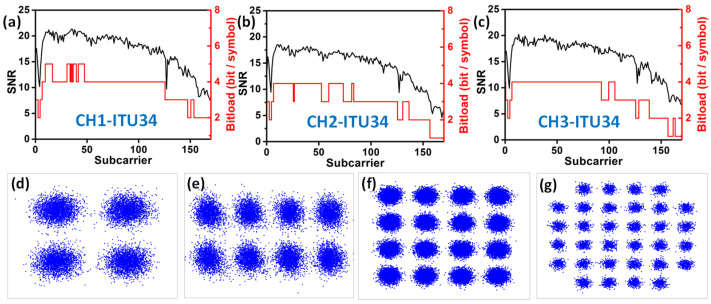
Experimental SNR and bit-loadings for each subcarrier in (**a**) CH1, (**b**) CH2 and (**c**) CH3 at the 1550.12 nm (ITU-34) wavelength channel. Typical constellations of (**d**) 4-QAM, (**e**) 8-QAM, (**f**) 16-QAM, (**g**) 32-QAM.

To increase the spectral efficiency of each DWDM wavelength channel, we apply bit-loadings to different OFDM subcarriers. Figure 9a–c show the signal-to-noise ratio (SNR) and bit-loadings for each subcarrier in different channels at the 1550.12 nm (ITU-34) wavelength channel. We can see that there is a notch near the 4th subcarrier, as shown in Figure 9a–c, and this is introduced by our electrical amplifiers. The highest bit-loading of 5 can be achieved, which corresponds to 32-quadrature amplitude modulation (QAM). The SNR and bit-loadings drops at the high frequency region are due to the bandwidth limitation of our RTO, and not to the SiPh 3 × 3 switch. Figure 9d,e illustrate the typical constellation diagrams of 4-QAM, 8-QAM, 16-QAM and 32-QAM of the ITU-34 wavelength channel. According to our experimental results, the average data rates of CH1, CH2 and CH3 are 58.71 Gbit/s, 46.64 Gbit/s and 53.65 Gbit/s, respectively. The total capacity of this 3 × 3 mode switch can achieve 7.623 Tbit/s (i.e., 53 Gbit/s × 3 modes × 48 wavelengths).

**Figure 10:**
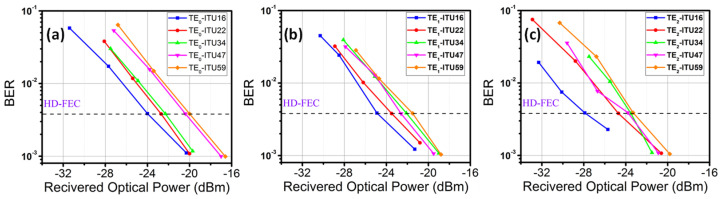
Measured BER curves of some selected wavelength channels of the 3 modes 3 dB optical power divider at (**a**) TE_0_, (**b**) TE_1_ and (**c**) TE_2_ modes.

**Figure 11:**
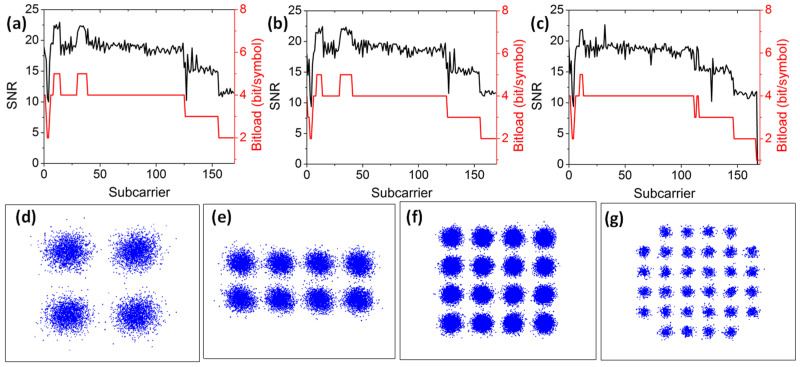
Experimental SNR and bit-loadings for each subcarrier at the power divided (**a**) TE_0_, (**b**) TE_1_ and (**c**) TE_2_ modes at the 1550.12 nm (ITU-34) wavelength channel. Typical constellations of (**d**) 4-QAM, (**e**) 8-QAM, (**f**) 16-QAM, (**g**) 32-QAM.

Finally, we experimentally characterize the MDM optical power divider. We evaluate the BER performances of all the 48 DWDM wavelength channels, and the BER performances after power divisions of TE_0_, TE_1_, and TE_2_ modes are shown in Figure 10a–c, respectively. Similarly, we select 1530.33 nm (ITU-16), 1539.77 nm (ITU-22), 1550.12 nm (ITU-34), 1559.79 nm (ITU-47) and 1564.68 nm (ITU-59) for display. We measure that every wavelength channel can satisfy the HD-FEC threshold. We also utilize the bit-loadings for different OFDM subcarriers to improve the spectral efficiency. Figure 11a–c show the SNR and bit-loadings for each subcarrier in different channels at the 1550.12 nm (ITU-34) wavelength channel. According to our experimental results, the average data rates of TE_0_, TE_1_ and TE_2_ mode division are 59.62 Gbit/s, 58.79 Gbit/s and 52.69 Gbit/s, respectively. The total capacity of this 3-dB MDM optical power divider can achieve 8.211 Tbit/s (i.e., 57.02 Gbit/s × 3 modes × 48 wavelengths). To summarize our experiment, we can observe that the 3 × 3 modes switch can achieve a total capacity of 7.623 Tbit/ss, while the 3-mode 3 dB power divider can achieve a total capacity of 8.211 Tbit/s. Hence, the whole system will be limited by the switch, and the expected total capacity of the whole system is 7.623 Tbit/s.

## 4. Conclusions

Combining MDM, WDM and OFDM at the same time can significantly increase the transmission capacity for optical interconnects. In this work, we proposed, fabricated and demonstrated a wideband and channel switchable MDM optical power divider on an SOI platform, supporting up to triple modes. The switchable MDM power divider consisted of two parts. The first part was a cascaded 3 × 3 MZI switch for switching the data from their original TE_0_, TE_1_ and TE_2_ modes to different modes among themselves. Then, mode up-conversion and Y-branch were utilized in the second part for the MDM power division. An average data rate of 57.02 Gbit/s was achieved in each wavelength. Here, a total capacity of 7.682 Tbit/s (i.e., 57.02 Gbit/s × 3 modes × 48 wavelengths) was achieved, satisfying the pre-HD-FEC threshold (BER = 3.8 × 10^−3^). The average data rate of each OFDM channel was limited by our RTO. Furthermore, although up to three MDM modes were presented in this proof-of-concept demonstration here, the proposed scheme can be scaled to higher order modes operation.

## Figures and Tables

**Table 1:** Performance of a single 2 × 2 switch.

Bias Voltage (V)	Measured Current (mA)	Calculated Resistance (kΩ)	Power Consumption (W)
5	3.754	1.332	0.019
10	7.421	1.348	0.074
12	9.062	1.324	0.109
15	10.99	1.365	0.165
20	14.316	1.397	0.286

## Data Availability

The data presented in this study are available from the first author upon request.
